# Dietary seaweed intake and depressive symptoms in Japanese adults: a prospective cohort study

**DOI:** 10.1186/s12937-019-0486-7

**Published:** 2019-10-07

**Authors:** Feng Guo, Cong Huang, Yufei Cui, Haruki Momma, Kaijun Niu, Ryoichi Nagatomi

**Affiliations:** 1 Department of Physical Education, Huaiyin Institute of Technology, Huaian, 223003 China; 2 Department of Medicine and Science in Sports and Exercise, Tohoku University Graduate School of Medicine, Sendai, 980-8575 Japan; 3 Department of Sports and Exercise Science, Zhejiang University, 148 Tianmushan Road, Xihu, Hangzhou, 310007 China; 4 Division of Biomedical Engineering for Health and Welfare, Tohoku University Graduate School of Biomedical Engineering, 2-1 Seiryo-machi, Aoba-ku, Sendai, 980-8575 Japan; 5 Department of Epidemiology, School of Public Health, Tianjin Medical University, 22 Qixiangtai Road, Heping District, Tianjin, 300070 China

**Keywords:** Seaweed consumption, Depressive symptoms, Longitudinal change, Employee

## Abstract

**Background:**

This prospective cohort study aimed to investigate the association between daily seaweed intake and depressive symptoms.

**Methods:**

In a prospective study conducted between 2008 and 2011, 500 Japanese adult employees aged 20–74 years participated and were included in the final analysis. Consumption of seaweed was assessed using a brief self-administered diet history questionnaire, and changes in seaweed consumption were divided into three categories (decreased, unchanged, and increased). Depressive symptoms were assessed using a Japanese version of the Self-Rating Depression Scale (SDS). Depressive symptoms were defined as an SDS score of ≥50 in the present study.

**Results:**

At the 3-year follow-up, 46 participants (9.2%) showed depressive symptoms. Multivariate analysis showed that baseline seaweed intakes were not significantly associated with the incidence of depressive symptoms (*p* for trend = 0.501). Conversely, odds ratios (95% CI) for depressive symptoms were lower in the participants who had higher seaweed intake than in those who had lower seaweed intake (decreased, 1.00; unchanged, 0.32 [0.13–0.81]; increased, 0.34 [0.13–0.88]; *p* for trend = 0.032) after adjusting for confounding factors.

**Conclusions:**

This study revealed a relationship between higher seaweed intake and a lower incidence of depressive symptoms in Japanese adults.

## Introduction

Depression is a common mental disorder, and the incidence of depressive illness has been steadily increasing over the past two decades [1]. The World Health Organization (WHO) reported that an estimated 350 million people of all ages worldwide have depression. Furthermore, according to WHO estimates, depression is the leading cause of disability and will become a leading factor in the global disease burden by the year 2030 [2]. Although effective treatments are available for depression, evidence shows that the recurrence rate for major depressive disorders in the general population is 23.2% after 10 years and 42.0% after 20 years of treatment [3]. Given this high recurrence rate for depressive illness, prevention seems more important than treatment to avoid depression.

Depressive symptoms are commonly affected by lifestyle. Examining the possible effects of lifestyle on depressive symptoms is necessary because most lifestyle factors are modifiable. The potential contributions of physical inactivity [4], smoking [5], and alcohol use [6] to relieve depressive symptoms have been well-demonstrated. Recently, some studies have focused on the association between diet and depressive symptoms and indicated that a healthy Japanese dietary pattern, characterized by a high intake of vegetables, mushrooms, soy products, and seaweeds, was associated with a lower prevalence of depressive symptoms [7, 8]. Among these food items of the traditional Japanese dietary pattern, which includes traditional sea vegetables [9], seaweed has been considered as a nutrient-rich dietary source of minerals, vitamins, and dietary fiber [10, 11]. Moreover, seaweed is also a rich source of antioxidants and anti-inflammatory properties [11]. Thus, higher seaweed consumption will likely contribute to the association between a healthy Japanese dietary pattern and lower depressive symptoms. Only one previous study examined the association between seaweed consumption and depressive symptoms [12]. In this study, higher seaweed consumption was independently associated with a lower prevalence of depressive symptoms during pregnancy after adjusting for potential confounding factors. The results indicated that seaweed consumption may be inversely associated with the prevalence of depressive symptoms during pregnancy in Japanese women.

To our knowledge, no previous prospective studies have been conducted on the association between seaweed consumption or dietary patterns that include seaweed intakes and depressive symptoms. Therefore, the present study aimed to investigate prospectively the relationship between seaweed consumption and incidence of depressive symptoms.

## Methods

### Study population

This study is part of the Sendai Oroshisho Study, which is a prospective cohort study on the risk factors of lifestyle-related diseases, especially metabolic syndrome, arteriosclerosis, and mental disorders. The Sendai Oroshisho Study was conducted at the Sendai Oroshisho Center, a group of over 120 small and medium enterprises in Sendai City, Japan, between 2008 and 2011. The study subjects participated in an annual health examination conducted every August, which included answering a questionnaire regarding depressive symptoms, dietary consumption, lifestyles, and socioeconomic status. Details of the study were reported previously [13].

**Fig. 1:**
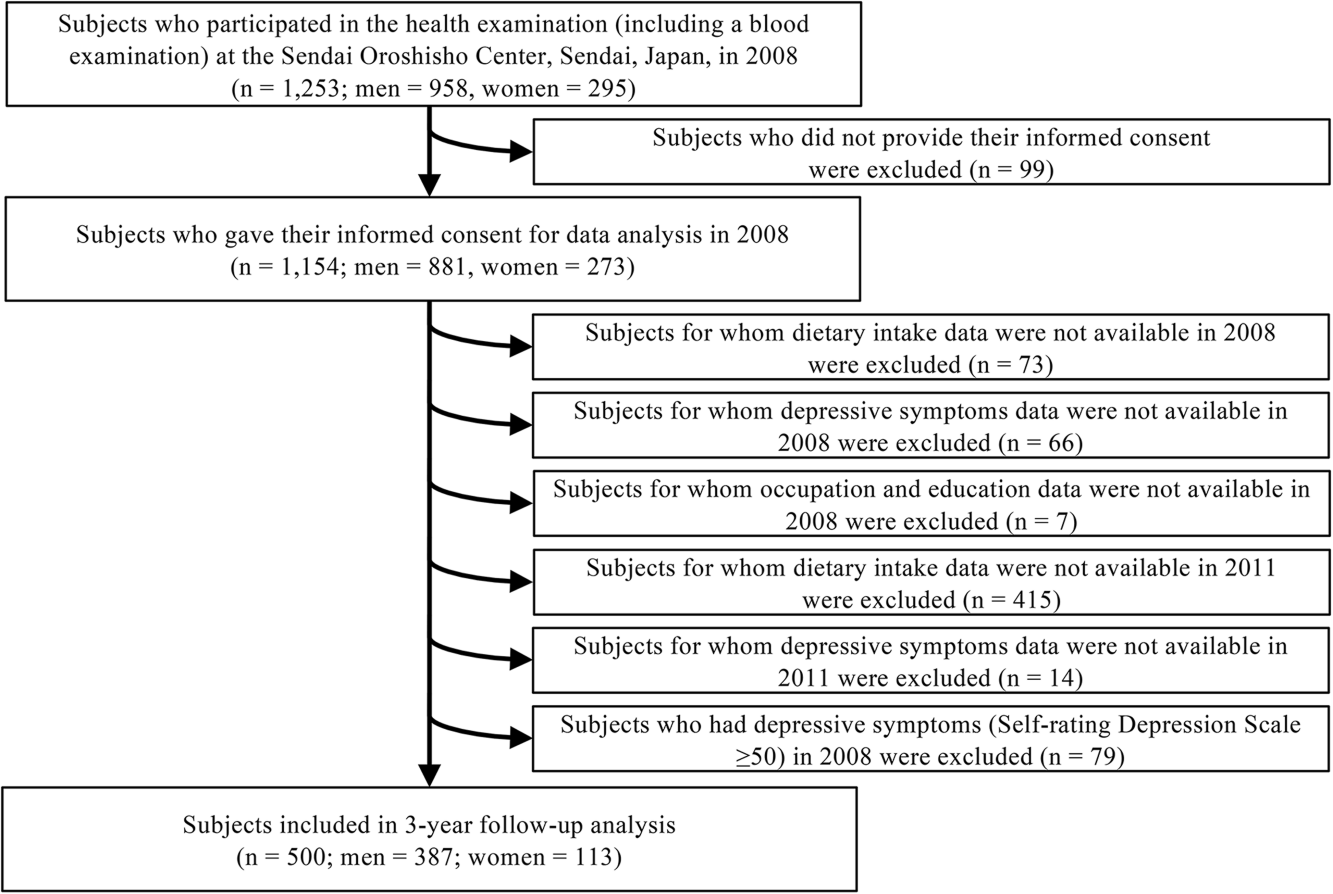
Flow chart for selection of study participants

Data from 1253 participants in 2008 were used as baseline data. Of the participants, 1154 provided written informed consent to participate in the study (response rate, 92.1%). Furthermore, participants were excluded if they met the following criteria: 1) no available information on depressive symptoms, dietary consumption, and lifestyles at baseline (*n* = 146); 2) no available information on depressive symptoms and dietary consumption at follow-up (*n* = 429); and 3) had depressive symptoms, defined as a Self-Rating Depression Scale (SDS) index score of ≥50 [14] at baseline (*n* = 79). After applying the exclusion criteria, 500 participants were included in the analysis (Fig. 1). The protocol for the present study was approved by the Institutional Review Board of the Tohoku University Graduate School of Medicine.

### Seaweed and other dietary intakes

A brief self-administered diet history questionnaire (BDHQ) that included questions on 75 food items along with their specified serving sizes was used to assess the dietary intakes of the preceding month [15]. For seaweed and other food items, the participants indicated their mean frequency of consumption in terms of the specified serving size by checking 1 of 7 frequency categories ranging from “almost never” to “2 or more times a day.” Estimates of mean daily intakes of energy, seaweed, green leafy vegetables, and fatty fish were calculated using an ad hoc computer algorithm for the BDHQ, which was based on the Standard Tables of Food Composition in Japan (5th edition) [16]. The validity of the BDHQ was reported elsewhere [17].

Further, energy-adjusted values by the density method (/1000 kcal) were used for all food items. Daily seaweed intake was summarized in tertiles based on its distribution (tertile 1, 0–2.9 g/1000 kcal/day; tertile 2, 2.9–7.3 g/1000 kcal/day; tertile 3, 7.3 − 46.1 g/1000 kcal/day). In order to examine the 3-year change in seaweed intake, the estimated daily seaweed intake was divided into 3 categories as follows: decreased group (decreased by < 2 g/1000 kcal/day), unchanged group (changed from − 2 to 2 g/1000 kcal/day), and increased group (increased by > 2 g/1000 kcal/day). Owing to the absence of a cutoff range to define “unchanged,” we also used some other cutoff values such as ±1 and ± 3 g/1000 kcal/day.

### Depressive symptoms

Depressive symptoms were assessed using the 20-item SDS, which has been used with various patients and healthy people [14]. Each item is rated on a scale of 1 to 4, with higher scores representing greater symptom severity. The total SDS scores ranged from 20 to 80. An SDS score < 50 was considered to be within the normal range, while an SDS score ≥ 50 was related to depressive symptoms [14]. SDS is considered as a useful instrument with high validity and sensitivity to evaluate depressive symptoms [18]. SDS was equally sensitive in differentiating mild from moderate compare with the Hamilton Rating Scale for Depression, but less sensitive in separating none from mild and moderate from severe. Although the sensitivity of SDS less than that of the Hamilton Rating Scale for Depression, it was found to be adequate [18]. On the other hand, SDS had good validity (coefficient alpha) with 0.90 and 0.86, respectively. SDS had better superiority than some other scales, such as the Beck Depression Inventory and the Minnesota Multiphasic Personality Inventory Depression scale, in male adults [19].

### Assessment of other variables

Sex, age, and socioeconomic status such as occupation (desk work or not), educational level (≥college or not), and living status (living alone or with others) were measured using a self-administered questionnaire. Lifestyle factors, including smoking status (current, former, or never), alcohol use frequency (every day, sometimes, or never), and breakfast skipping (skip ≥3 times/week or not) were also assessed using a self-report questionnaire. Moreover, physical activity was assessed using the International Physical Activity Questionnaire [20]. Total physical activity was calculated and categorized into three groups as follows: 0, 0.1–22.9, and ≥ 23 metabolic equivalent (MET) hours/week [21]. Anthropometric factors (height, weight, and waist circumference) were measured with a standardized protocol. Body mass index (BMI) was calculated as weight in kilograms divided by the square of height in meters (kg/m^2^). Systolic and diastolic blood pressures were measured from the upper right arm using an automatic device (Yamasu 605P; Kenzmedico, Saitama, Japan) after several minutes in a seated position. Under fasting conditions, blood samples were drawn from the antecubital veins after a resting period in a seated position. Concentrations of serum glucose (Eurotec, Tokyo, Japan), triglycerides, and high-density lipoprotein-cholesterol (Sekisui Medical, Tokyo, Japan) were analyzed with enzymatic methods using the appropriate kits. Metabolic syndrome was defined according to the criteria of the American Heart Association’s Scientific Statement of 2009 for people of Asian ethnicity [22].

### Statistical analysis

In the present study, data were presented as means (standard deviation) for continuous variables and percentages or odds ratios (95% confidence interval [CI]) for categorical variables. The association between the participants’ characteristics and the seaweed intakes was tested using the analysis of variance or the chi-square test for trend. Multivariate logistic regression analysis was used to examine the association between daily seaweed intake and incidence of depressive symptoms. All continuous variables at baseline were log-transformed prior to the analysis because their distribution was skewed. Depressive symptoms were used as the dependent variables, and the baseline seaweed intake or change in seaweed intake was used as the independent variable. Covariates included baseline SDS scores, baseline seaweed intake (if necessary), sex, log age, and log BMI in model 1. Model 2 was additionally adjusted for socioeconomic status (occupation, educational levels, and living status) at baseline. For model 3, all of the variables in models 1 and 2 were used, in addition to the baseline lifestyle factors and health status, including smoking status, alcohol use frequency, breakfast skipping, physical activity, and metabolic syndrome. As appropriate, model 4 was additionally adjusted for baseline green leafy vegetable intake or change in green leafy vegetable intake and baseline fish intake or change in fish intake. The odds ratios and 95% CI of the incidence of depressive symptoms compared with the lowest frequency of seaweed intake or decreased intake of the seaweed category as the reference were calculated to test the *p* value for linear trend. The interactions between the change in seaweed intake and all the covariates for incident depressive symptoms were examined through the addition of the cross-product terms to the multivariate logistic regression analysis model. All the statistical tests were two-tailed, and *p* values of < 0.05 were considered statistically significant. Analyses were performed using the IBM SPSS Statistics 22.0 software for Mac (IBM Corp, Armonk, NY, USA).

## Results

### Participant characteristics

The participants’ ages ranged from 20 to 74 years, with a mean (SD) of 45.7 (10.2) years. Of the participants, 113 were women, which accounted for 22.6% of the study population. The participants’ characteristics, according to the frequency of seaweed intake, are presented in Table 1. Frequency of seaweed intake was positively associated with age, and intakes of protein, green leafy vegetables, and fish (*p* ≤ 0.001). The proportion of men, low educational level, living alone, and skipping breakfast habit was higher in the group with low seaweed category intake (*p* < 0.05). Furthermore, smoking, alcohol use, and SDS scores were inversely associated with the frequency of seaweed intake, with a borderline significance. No association was found between the frequency of seaweed intake and other factors.

**Table 1:** Participants’ characteristics according to the tertiles of seaweed intakes at baseline (*n* = 500)

Variables	Tertiles of seaweed intakes			*p* value^a^
	Tertile 1	Tertile 2	Tertile 3	
Seaweed intakes, g/1000 kcal/day	1.6 (0.9)	5.0 (1.4)	13.1 (6.3)	
No. of participants	167	167	166	
Sex (women), %	20.4	18.0	29.5	0.046
Age, years	44.0 (10.0)	45.4 (9.6)	47.6 (10.6)	0.001
BMI, kg/m^2^	23.2 (3.3)	23.2 (3.9)	23.0 (3.3)	0.530
Education (≥college), %	21.6	33.5	31.9	0.037
Occupation (desk work), %	46.1	43.7	48.8	0.624
Living status (alone), %	15.0	9.0	7.2	0.021
Smoking status				
Current, %	46.1	46.1	38.6	
Former, %	15.6	13.2	13.9	
Never, %	38.3	40.7	47.6	0.098
Alcohol intake frequency				
Every day, %	31.7	25.7	27.1	
Sometimes, %	48.5	56.3	45.8	
Never, %	19.8	18.0	27.1	0.120
Physical activity				
0, MET hours/week	24.0	22.2	24.1	
0.1–22.9, MET hours/week	38.9	38.3	40.4	
≥ 23, MET hours/week	37.1	39.5	35.5	0.838
Skipping breakfast, %	29.3	20.4	13.9	0.001
Metabolic syndrome, %	18.6	14.4	18.7	0.980
Protein intakes, g/1000 kcal/day	32.8	34.1	36.4	< 0.001
Green leafy vegetables intakes, g/1000 kcal/day	15.2 (16.4)	16.5 (13.0)	24.4 (19.5)	< 0.001
Fish intakes, g/1000 kcal/day	15.2 (11.8)	16.2 (9.4)	19.9 (12.7)	< 0.001
SDS scores	38.9 (6.6)	39.0 (6.8)	37.5 (7.5)	0.077

Data are presented as mean (standard deviation), or percentage

*BMI* Body mass index, *MET* Metabolic equivalent, *SDS* Self-rating depression scale

^a^Differences were evaluated using analysis of covariance and chi-squared test for linear trend, as appropriate

### Association between dietary seaweed intake and depressive symptoms

Among the participants without depressive symptoms in 2008, 46 (9.2%) had incident depressive symptoms in 2011. Multivariate regression analysis revealed that the baseline seaweed intake tertiles were not linearly associated with incident depressive symptoms after adjusting for all potential confounding factors (*p* = 0.501), while the incidence of depressive symptoms was higher in the second tertile of seaweed intake than in the first tertile of seaweed at baseline (*p* = 0.046; Table 2). On the other hand, the 3-year change in seaweed intake was shown to be associated with the incidence of depressive symptoms (Table 3). In model 3, the incidence of depressive symptoms was lower in the group with higher seaweed intake than in the group with lower intake when adjusted for baseline SDS scores, sex, log age, log BMI, occupation, educational levels, living status, smoking status, alcohol use frequency, breakfast skipping, physical activity, and metabolic syndrome (*p* for trend = 0.022). After additionally adjusting for change in the intakes of protein, green leafy vegetables, and fish, the odds ratios for the incidence of depressive symptoms were still high in the groups with lower seaweed intake (*p* for trend = 0.032). A sensitivity analysis showed similar results when the “unchanged” category was defined as ±1 or ± 3 g/1000 kcal/day (*p* for trend < 0.05) (Table 4). There was no interaction between the change in seaweed intake and baseline SDS scores with the incidence of depressive symptoms (data not shown).

**Table 2:** Multivariate logistic regression of the odds ratios (95% confidence interval) for the incidence of depressive symptoms by tertiles of seaweed intakes at baseline (*n* = 500)

	Tertiles of seaweed intakes at baseline			*p* for trend
	Tertile 1	Tertile 2	Tertile 3	
Range, g/1000 kcal/day	0, 2.9	2.9, 7.3	7.3, 46.1	
No. of participants	167	167	166	
No. of participants with depressive symptoms	10	21	15	
Model 1^a^	1.00 (ref)	2.23 (0.99–5.01)	1.72 (0.73–4.09)	0.226
Model 2^b^	1.00 (ref)	2.27 (0.99–5.19)	1.75 (0.72–4.22)	0.229
Model 3^c^	1.00 (ref)	2.51 (1.07–5.93)	1.51 (0.61–3.77)	0.399
Model 4^d^	1.00 (ref)	2.43 (1.02–5.82)	1.41 (0.55–3.65)	0.501

^a^Adjusted for baseline Self-rating Depression Scale, sex, log age, and log body mass index

^b^Same as Model 1 + occupation (desk work), education level (≥college), and living status (alone)

^c^Same as Model 2 + smoking status (current, former, never), alcohol use frequency (every day, sometimes, never), breakfast skipping (≥3 times/week), physical activity (0, 0.1–22.9, ≥ 23 metabolic equivalent hours/week), and metabolic syndrome

^d^Same as Model 3 + log intakes of protein, green leafy vegetables, and fish

**Table 3:** Multivariate logistic regression of the odds ratios (95% confidence interval) for the incidence of depressive symptoms by change in seaweed intakes at 3-year follow-up (*n* = 500)

	Change in seaweed intakes			*p* for trend
	Decreased	Unchanged	Increased	
Range, g/1000 kcal/day	–46.1, −2.0	−2.0, 2.0	2.0, 39.5	
No. of participants	152	193	155	
No. of participants with depressive symptoms	21	14	11	
Model 1^a^	1.00 (ref)	0.42 (0.18–0.97)	0.39 (0.16–0.94)	0.043
Model 2^b^	1.00 (ref)	0.32 (0.13–0.78)	0.33 (0.13–0.82)	0.025
Model 3^c^	1.00 (ref)	0.33 (0.13–0.83)	0.32 (0.13–0.81)	0.022
Model 4^d^	1.00 (ref)	0.32 (0.13–0.81)	0.34 (0.13–0.88)	0.032

^a^Adjusted for baseline Self-rating Depression Scale, baseline seaweed intakes, sex, log age, and log body mass index

^b^Same as Model 1 + occupation (desk work), education level (≥college), and living status (alone)

^c^Same as Model 2 + smoking status (current, former, never), alcohol use frequency (every day, sometimes, never), breakfast skipping (≥3 times/week), physical activity (0, 0.1–22.9, ≥ 23 metabolic equivalent hours/week), and metabolic syndrome

^d^Same as Model 3 + change in intakes of protein, green leafy vegetables, and fish

**Table 4:** Multivariate logistic regression of the odds ratios (95% confidence interval) for the incidence of depressive symptoms by change in seaweed intakes with different cutoff values at 3-year follow-up (*n* = 500)

	Change in seaweed intakes			*p* for trend
	Decreased	Unchanged	Increased	
Range, g/1000 kcal/day	−46.1, −1.0	−1.0, 1.0	1.0, 39.5	
No. of participants	193	117	190	
No. of participants with depressive symptoms	24	9	13	
Model 1^a^	1.00 (ref)	0.55 (0.22–1.36)	0.47 (0.21–1.03)	0.063
Model 2^b^	1.00 (ref)	0.44 (0.17–1.11)	0.41 (0.18–0.92)	0.035
Model 3^c^	1.00 (ref)	0.46 (0.18–1.21)	0.37 (0.16–0.87)	0.025
Model 4^d^	1.00 (ref)	0.47 (0.18–1.26)	0.40 (0.17–0.94)	0.037
	Change in seaweed intakes			*p* for trend
	Decreased	Unchanged	Increased	
Range, g/1000 kcal/day	−46.1, −3.0	−3.0, 3.0	3.0, 39.5	
No. of participants	120	252	128	
No. of participants with depressive symptoms	15	25	6	
Model 1^a^	1.00 (ref)	0.70 (0.29–1.67)	0.29 (0.10–0.91)	0.029
Model 2^b^	1.00 (ref)	0.55 (0.22–1.36)	0.29 (0.08–0.82)	0.020
Model 3^c^	1.00 (ref)	0.57 (0.22–1.47)	0.23 (0.07–0.77)	0.015
Model 4^d^	1.00 (ref)	0.61 (0.23–1.60)	0.27 (0.08–0.89)	0.023

^a^Adjusted for baseline Self-rating Depression Scale, baseline seaweed intakes, sex, log age, and log body mass index

^b^Same as Model 1 + occupation (desk work), education level (≥college), and living status (alone)

^c^Same as Model 2 + smoking status (current, former, never), alcohol use frequency (every day, sometimes, never), breakfast skipping (≥3 times/week), physical activity (0, 0.1–22.9, ≥ 23 metabolic equivalent hours/week), and metabolic syndrome

^d^Same as Model 3 + change in intakes of protein, green leafy vegetables, and fish

## Discussion

In this prospective study of seaweed intake and depressive symptoms, increased intake of seaweed was associated with a decreased incidence of depressive symptoms at the 3-year follow-up, after adjusting for sex, age, socioeconomic status, lifestyle factors, and other dietary intakes, although multivariate analysis revealed that the baseline daily seaweed intake did not predict the presence of depressive symptoms at follow-up.

The findings of our study support the recent cross-sectional report that showed a high frequency of seaweed consumption was associated with low prevalence of depressive symptoms in pregnant women [12]. This previous study is the only study to date that examined the independent association between seaweed intake and depressive symptoms. In addition, the results of the two other cross-sectional studies with Japanese employees partially agreed with these results, in which a healthy dietary pattern characterized by vegetables, mushrooms, soy products, and seaweeds was associated with a low prevalence of depressive symptoms [7, 8]. However, the independent association of seaweed intake with depressive symptoms was not reported in the two previous studies. To the best of our knowledge, our study represents the first evidence of the prospective association between seaweed intake and depressive symptoms.

It is interesting that the incidence of depressive symptoms was associated with changes in seaweed intake but not with baseline seaweed intake. Some studies indicated that dietary habits were relatively stable in adults [23, 24], suggesting that dietary patterns identified in the recent past may provide useful information about current dietary patterns. However, Mulder et al. [25] demonstrated that dietary habits had great variability over a 4-year period, which is similar to the follow-up period of the present study. This implies that dietary habit is not a unified concept. In general, people tend to eat healthier with increasing age.

It is important to note that some participants may have changed their dietary habits owing to the 2011 Great East Japan Earthquake in March 2011, which occurred 5 months before the follow-up investigation. Although we cannot directly prove this hypothesis, we considered that, generally, Japanese people might have chosen to eat less seafood than before, given the risk that the Fukushima nuclear accident contaminated agricultural and fishery products. Among these participants, it is considered that the most susceptible may have been those who had a high consumption of seaweed at baseline. Meanwhile, there is another possibility that participants may have consumed more seaweed after the earthquake and Fukushima nuclear accident to increase the intake of iodine-127, which was considered to have a therapeutic effect on thyroid diseases [26]. Thus, in the current study, seaweed intake might have changed during the 3-year follow-up period, and the changes in seaweed intake reflect the current trends of dietary habits.

It is hypothesized that the inverse association between changes in seaweed intake and depressive symptoms may be explained by many nutrients contained in seaweed, including folate, vitamin B6, vitamin B12, and n-3 polyunsaturated fatty acids (PUFAs). In fact, depressive symptoms have been considered to have a neurochemical basis, such as the monoaminergic neurotransmitters system [27]. Folate and vitamins B6 and B12 are involved in the mechanism of monoamines such as serotonin and other monoamine neurotransmitters [28, 29]. The n-3 PUFAs have been associated with the dynamic structure of the central nervous system neuronal membranes by increasing their fluidity and serotonin transport [30, 31]. The potential neuroprotective effects of these nutrients have been supported by the results of some population-based studies. Lower intakes of folate and vitamins B6 and B12 at baseline were associated with a higher risk of depressive symptoms at follow-up [32, 33]. A preliminary double-blind, placebo-controlled trial reported that n-3 PUFAs could improve the short-term course of illness and were well tolerated in patients with major depressive disorders [34].

This study has some limitations. First, given the possibility that a change of seaweed intake may have been a result of the 2011 Great East Japan Earthquake, the impact of the disaster could be considered as a potential confounding factor. Second, information on the species of seaweeds was not available. In fact, different species of seaweeds may have different impacts on depressive symptoms because the nutritional value of seaweeds differs according to species and family [9]. Third, the dietary questionnaire we used did not confirmed cooking methods of seaweed, these potential qualitative differences of seaweed may affect the observed results. Four, causality could not be inferred in our data due to the observational nature of the study design.

## Conclusion

In conclusion, the results of this prospective study of Japanese adults indicated that increased seaweed intake was associated with a decreased incidence of depressive symptoms, independent of sex, age, BMI, socioeconomic status, lifestyle factors, and intakes of other food items. This study is the first to investigate the prospective relationship between seaweed intake and depressive symptoms. Randomized controlled trials and experimental studies are warranted to investigate this association and clarify the underlying mechanism between seaweed intake and depressive symptoms.

## Data Availability

Not applicable.

## Abbreviations

BDHQ: Brief self-administered diet history questionnaire
BMI: Body mass index
MET: Metabolic equivalent
PUFAs: Polyunsaturated fatty acids
SDS: Self-rating depression scale
WHO: World health organization
